# Perceived Availability of Healthy and Unhealthy Foods in the Community, Work, and Higher Education Settings across Five Countries: Findings from the International Food Policy Study 2018

**DOI:** 10.1093/jn/nxac070

**Published:** 2022-05-11

**Authors:** Alejandra Contreras-Manzano, Claudia Nieto, Alejandra Jáuregui, Carolina Pérez Ferrer, Lana Vanderlee, Simón Barquera, Gary Sacks, Jean Adams, James F Thrasher, David Hammond

**Affiliations:** Center for Nutrition and Health Research, National Institute of Public Health, Cuernavaca, Mexico; Center for Nutrition and Health Research, National Institute of Public Health, Cuernavaca, Mexico; Center for Nutrition and Health Research, National Institute of Public Health, Cuernavaca, Mexico; Center for Nutrition and Health Research, National Institute of Public Health, Cuernavaca, Mexico; National Council for Science and Technology, Mexico City, Mexico; École de Nutrition, Centre Nutrition, santé et société (Centre NUTRISS), and Institut sur la nutrition et les aliments fonctionnels (INAF), Université Laval, Québec, Canada; Center for Nutrition and Health Research, National Institute of Public Health, Cuernavaca, Mexico; Global Obesity Centre (GLOBE), Institute for Health Transformation, Deakin University, Burwood Victoria, Geelong, Australia; Medical Research Council Epidemiology Unit, University of Cambridge, Cambridge, United Kingdom; Department of Health Promotion, Education and Behavior, Arnold School of Public Health, University of South Carolina, Columbia, SC, USA; School of Public Health and Sciences, University of Waterloo, Waterloo, Canada

**Keywords:** perceived availability, food policy, food environment, community, university, work environment

## Abstract

**Background:**

Food environments play a key role in dietary behavior and vary due to different contexts, regulations, and policies.

**Objectives:**

This study aimed to characterize the perceived availability of healthy and unhealthy foods in 3 different settings in 5 countries.

**Methods:**

We analyzed data from the 2018 International Food Policy Study, a cross-sectional survey of adults (18–100 y, *n* = 22,824) from Australia, Canada, Mexico, the United Kingdom (UK), and the USA. Perceived availability of unhealthy (junk food and sugary drinks) and healthy foods (fruit or vegetables, healthy snacks, and water) in the community, workplace, and university settings were measured (i.e. not available, available for purchase, or available for free). Differences in perceived availability across countries were tested using adjusted multinomial logistic regression models.

**Results:**

Across countries, unhealthy foods were perceived as highly available in all settings; in university and work settings unhealthy foods were perceived as more available than healthy foods. Australia and Canada had the highest perceived availability of unhealthy foods (range 87.5–90.6% between categories), and the UK had the highest perceived availability of fruits and vegetables for purchase (89.3%) in the community. In university and work settings, Mexico had the highest perceived availability for purchase of unhealthy foods (range 69.9–84.9%). The USA and the UK had the highest perceived availability of fruits and vegetables for purchase (65.3–66.3%) or for free (21.2–22.8%) in the university. In the workplace, the UK had high perceived availability of fruits and vegetables for purchase (40.2%) or for free (18.5%), and the USA had the highest perceived availability of junk food for free (17.3%).

**Conclusions:**

Across countries, unhealthy foods were perceived as highly available in all settings. Variability between countries may reflect differences in policies and regulations. Results underscore the need for the continuation and improvement of policy efforts to generate healthier food environments.

## Introduction

There is a growing epidemic of obesity and noncommunicable diseases related to diet (1). A key driver of this epidemic is the availability and accessibility of foods in the environments where people live, study, and work (2, 3). Over the last several years, the global demand and supply of unhealthy foods with salt, sugars, saturated fats, and *trans* fats has increased, often at the expense of diversity and displacing local and healthier diets (4). Few studies have compared food environments across countries due to a lack of comparable data. Identifying similarities and differences in food environments between countries has the potential to highlight drivers of country-level variability in dietary patterns, evaluate differences in policy approach, and identify opportunities for new policy interventions.

Food environments vary across countries due to different contexts, regulations, and policies implemented with various degrees of enforcement (4, 5) (**Supplemental Table 1**) (6–35). Food environment interventions in education centers are among the most common strategies used to limit access to unhealthy foods or increase the availability of healthier foods (36–39). However, these regulations are not always adopted in higher education settings. In some cases, universities have guidelines that focus on specific aspects of the food environment, which may include removing sugar-sweetened beverages, reducing portion sizes, pricing strategies, and increasing the accessibility to healthy choices (6, 40, 41).

Worksite food access policies are even more heterogeneous as they often respond to occupational health policies of public and private sector organizations, with few governmental programs in place (12). Guidelines for making healthier choices more available in these settings have been used in several countries (13, 16, 21, 42–44). However, guidelines are generally voluntary, except for a few cases where nutrition guidelines are mandatory (i.e. in UK central government contexts) (45).

The community food environment (e.g. location and accessibility of food outlets outside the home) is less regulated than schools or workplaces, with few interventions conducted at the local governmental level (15). The evidence around “what works” to foster healthy food environments at the community level is still developing; however, some options include zoning ordinances and land-use plans which can influence placement and access to food outlets, as well as in-store policies aiming to improve access to healthy foods (46, 47).

Although most studies investigating the food environment have used objective measures, perceived measures may play an important and distinct role in influencing diet, since they take into account the experience and reality for consumers (3, 48). Perceived measures of the food environment are correlated with objective measures and become relevant since they are a critical mediating factor with respect to consumer behaviors (47). Studies suggest that personal perceptions might be stronger determinants of food acquisition, diets, and health, than other objective measures like proximity (49).

To our knowledge, no previous study has examined the perceived availability of healthy and unhealthy foods in different settings across countries. This study aimed to describe the perceived availability of healthy and unhealthy foods in the community, workplace, and university settings across 5 countries. Based on global trends in food supply as well as differences in available efforts to regulate food environments across settings (4), we hypothesized that the perceived availability of unhealthy foods is higher than for healthy foods in all the countries analyzed, but may vary across community, workplace, and university settings and between countries.

## Methods

We analyzed data from the 2018 International Food Policy Study (IFPS), a cross-sectional survey of adults aged 18–100 y (*n* = 22,824) from 5 countries; Australia, Canada, Mexico, the UK, and the USA. The selection of countries was based on broad similarities in the food environment, language, and culture. In the case of Mexico, geographic proximity and sociocultural similarities to key US subpopulations were also a consideration. The IFPS countries also differ in major national-level nutrition policies that have been implemented, including marketing restrictions, food labeling, and fiscal policies.

Data were collected via self-completed web-based surveys conducted in November/December 2018. The study sample was recruited via Nielsen Consumer Insights Global Panel and their partners’ panels. Nielsen drew random samples from online panels in each country, stratified for age and sex utilizing quotas that approximated the known proportions for males and females in 4 age groups: 18–25, 30–44, 45–64, and 65–100 y, according to national census estimates. E-mail invitations with unique survey access links were sent to a random sample of panelists within each country after targeting for demographics; panelists known to be ineligible were not invited. Potential respondents were screened for eligibility and quota requirements using age, sex, and minimum device screen size (to restrict respondents from completing the survey on a smartphone). Surveys were conducted in English in Australia and the UK; Spanish in Mexico; English or French in Canada; and English or Spanish in the USA (based on the panelist's known language preference).

All potential participants were provided with information about the study and were asked to provide informed consent before completing the online survey. Participants received compensation by their panel's usual incentive structure (e.g. points-based or monetary rewards, chances to win prizes) after completing the survey. The study was reviewed by and received ethics clearance through a University of Waterloo Research Ethics Committee (ORE# 30,828). A full description of the study methods and the questionnaires can be found in the IFPS: Technical Report – 2018 Survey (Wave 2. Available at: at https://foodpolicystudy.com/methods/).

### Perceived availability of foods and beverages

The current study analyzed survey questions related to the perceived availability of healthy and unhealthy foods in the community, university, and/or work. Perceived *food availability in the community* (*n* = 21,369) was measured with the question: “Are the following foods or drinks sold in stores you can get to WITHIN 5 MINUTES FROM YOUR HOME, using your usual mode of transportation (e.g. walk, drive, or public transit)?” for the following foods or drink categories: junk food; fresh fruit or vegetables; other healthy snacks; sugary drinks; and clean drinking water. Response options were: not available to buy; available to buy; don't know; or refuse to answer. Perceived *food availability in universities* was examined among adults who reported attending an education center with the question (*n* = 3253), “Are the following foods or drinks available at your SCHOOL? Do not include items you bring from home,” with the same food and drink categories. Response options were: not available, available to buy, available for free, don't know, or refuse to answer. A similar question was used to assess perceived *food availability in workplaces* among those who reported working at a paid job or business (*n* = 11,233) with the same response options. Responses were recategorized as follows: 0 = Not available (Not available to buy or not available), 1 = Available for purchase, and 2 = Available for free. Participants answering don't know or refuse to answer, as well as those with discrepant responses (e.g. the participant indicated not available to buy and available to buy) were treated as missing data.

### Covariates

Demographic information was assessed using survey measures taken directly or adapted from population-level surveys within each country (50–55). Variables were recoded and harmonized for comparison across countries, and included sex at birth (male; female), age (continuous), education level was categorized as “low” (i.e. completed secondary school or less), “medium” (i.e. some postsecondary qualifications), or “high” (i.e. university degree or higher) according to country-specific criteria related to the highest level of formal education completed. Race or ethnicity was categorized as “majority” if participants only identified themselves as “white” in Canada, the UK, and the USA, solely English-speaking in Australia, or non-Indigenous in Mexico. Income adequacy was measured with the question: “Thinking about your total monthly income, how difficult or easy is it for you to make ends meet?” with responses collapsed into very difficult or difficult (“very difficult” and “difficult”), neither easy nor difficult, and easy or very easy (“Easy” and “Very easy”). Self-reported nutrition knowledge was assessed with the question “How would you rate your nutrition knowledge?” with responses collapsed into not knowledgeable (“not at all knowledgeable” and “a little knowledgeable”), somewhat knowledgeable, and knowledgeable (“very knowledgeable” and “extremely knowledgeable”).

### Data analysis

A total of 22,824 adults completed the survey. After removing participants who did not provide information about their perceived availability of foods and beverages at any of the settings (i.e. not answering or answering don't know, refuse to answer, or having discrepant responses for the university, work, or community) (*n* = 572), and those with invalid or missing responses for covariates (*n* = 486), a total of 21,766 were retained in the analyses (Canada: *n* = 4156; Australia: *n* = 3941; UK: *n* = 5181; USA: *n* = 4474; and Mexico: *n* = 4014). All analyses were weighted with poststratification sample weights constructed using a ranking algorithm with population estimates from the census in each country, based on age group, sex at birth, region, ethnicity (except in Canada), and education (except in Mexico).

To determine differences by sociodemographic characteristics and ethnicity, linear regression and Pearson χ2 tests were calculated. To compare the perceived availability of foods and beverages in the university, community, and work settings across countries, we fitted multinomial logistic regression models with the perceived availability as the outcome measure (0 = No availability, 1 = Available for free, 2 = Available for purchase) and the country as the independent variable. Separate models were fitted for each setting and food category. All models were adjusted for age, sex, race or ethnicity, education level, income adequacy, and self-reported nutritional knowledge. Comparisons between all countries and availability categories were examined by introducing each country or category as the reference category. Adjusted percentages and 99% CIs derived from multinomial logistic regression models were estimated and graphically presented. Analyses were conducted using Stata v14.

## Results

**FIGURE 1:**
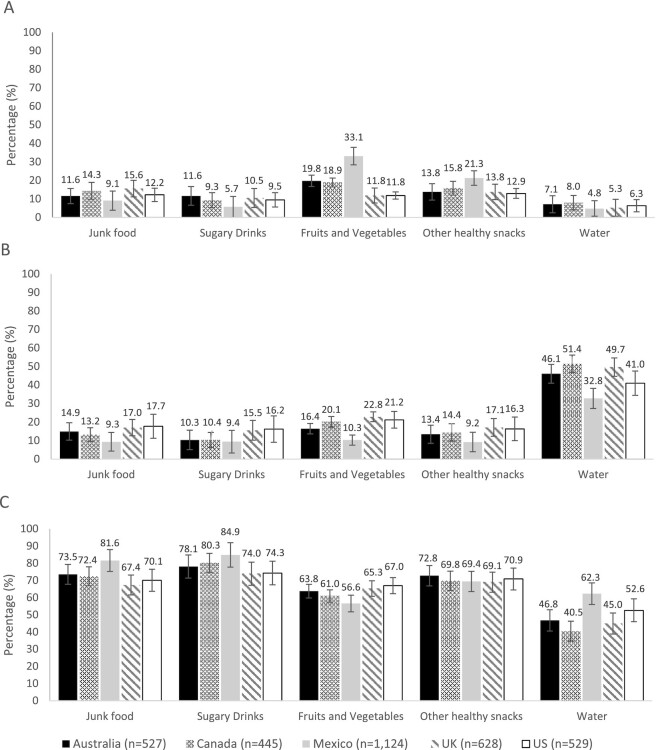
Adjusted percentage of participants reporting that different food and beverages are or are not available for purchase or for free in the university setting in all countries (IFPS 2018, *n* = 3295). All percentages were adjusted by age, sex, education level, race or ethnicity, income adequacy, and nutritional knowledge throughout multinomial logistic regression models. 99% CI. Panel A: not available, B: available for free, and C: available for purchase. International Food Policy StudyS.

**FIGURE 2:**
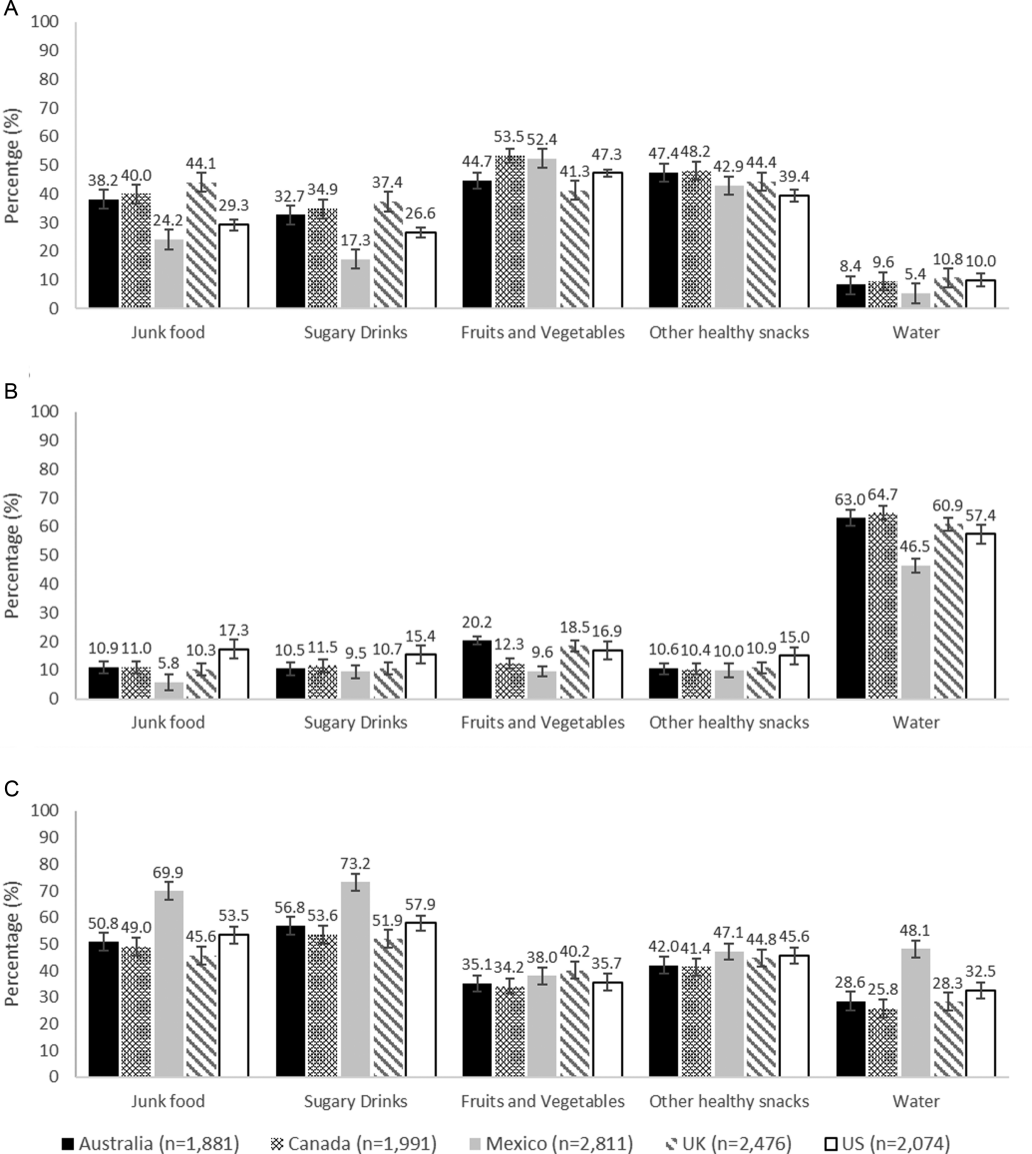
Adjusted percentage of participants reporting that different food and beverages are or are not available for purchase or for free in the workplace in all countries (IFPS 2018, *n* = 11,247). All percentages were adjusted by age, sex, education level, race or ethnicity, income adequacy, and nutritional knowledge throughout multinomial logistic regression models. 99% CI. Panel A: not available, B: available for free, and C: available for purchase. International Food Policy Study.

**FIGURE 3:**
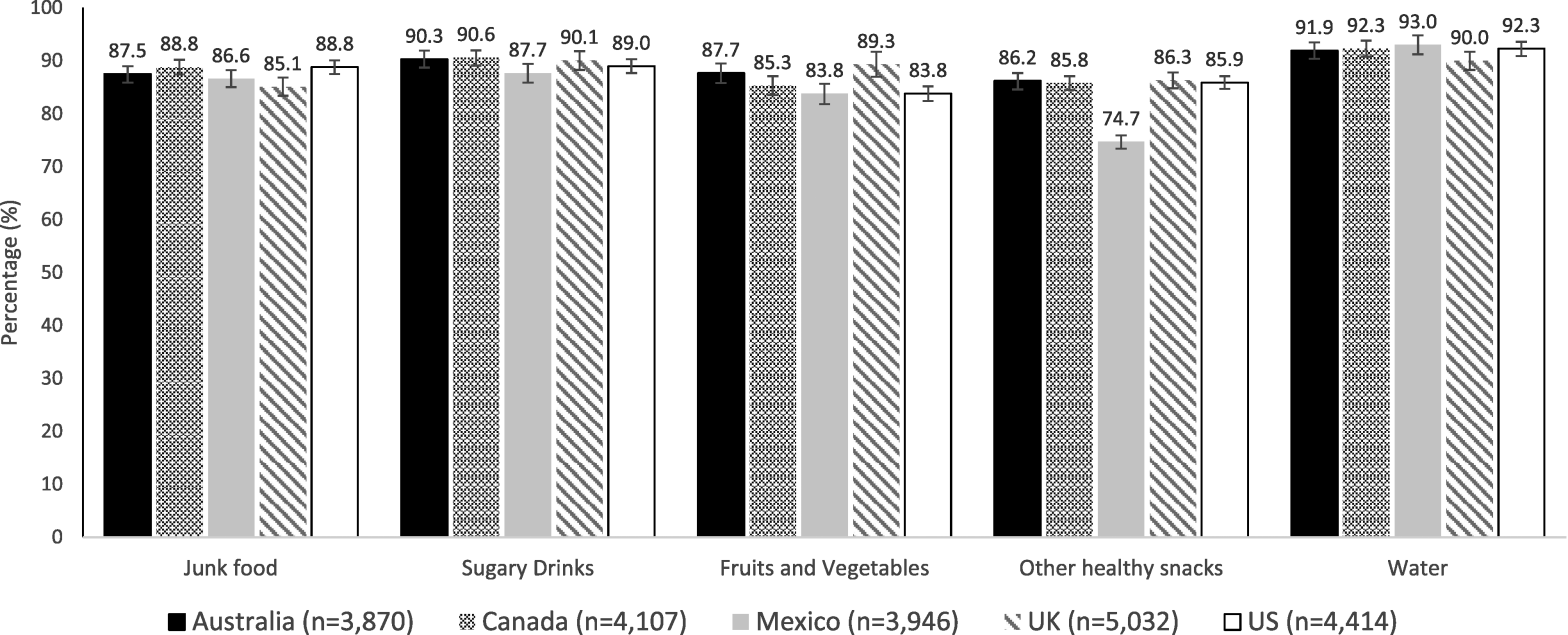
Adjusted percentage of participants reporting that different food and beverages are available for purchase in the community setting in all countries (IFPS 2018, *n* = 21,791). All percentages were adjusted by age, sex, education level, race or ethnicity, income adequacy, and nutritional knowledge throughout multinomial logistic regression models. 99% CI. International Food Policy StudyS.

All sample characteristics, except sex, were significantly different among countries (*P* <0.05) (**Table 1**). In all settings, the perceived availability of sugary drinks and junk food across countries was greater than the perceived availability of fruits and vegetables and other healthy foods (**Figures 1**–**3**). Differences in the availability of each food category were observed across countries in the university (**Supplemental Table 2**), workplace (**Supplemental Table 3**), and the community (**Supplemental Table 4**). These differences are discussed in the following sections and, for ease of interpretation, in some cases relative risk ratios are presented using a different reference category (country or availability category) than the one presented in the Supplementary Tables.

**TABLE 1:** Sociodemographic characteristics of participants of the study (IFPS 2018, *n* = 21,766)^1^

		All countries	Australia	Canada	Mexico	UK	USA	
	*n* sample	21,766	3941	4156	4014	5181	4474	*P* values
		Mean (99% CI)^2^						
	Age, y	46.0 (45.7, 46.3)	46.6 (45.8, 47.4)	48.4 (47.5, 49.2)	39.5 (38.7, 40.2)	47.9 (47.2, 48.6)	47.0 (46.2, 47.8)	<0.001
		% (99% CI)^2^						
Sex	Males	48.8 (47.8, 49.8)	48.9 (46.6, 51.2)	49.9 (47.5, 2.3)	47.6 (45.2, 50.0)	49.2 (47.7, 51.3)	48.4 (46.1, 50.8)	0.483
	Females	51.2 (50.1, 52.2)	51.1 (48.7, 53.3)	50.1 (47.7, 52.5)	52.4 (49.9, 54.8)	50.8 (48.6, 52.9)	51.6 (49.2, 53.9)	
Ethnicity	Majority	80.1 (79.2, 81.0)	75.7 (73.2, 78.1)	79.5 (77.5, 81.3)	78.7 (76.3, 80.9)	88.9 (87.3, 90.3)	75.7 (73.7, 77.7)	<0.001
	Minority	19.9 (18.9, 20.8)	24.3 (21.9, 26.7)	20.5 (18.6, 22.5)	21.3 (19.1, 23.7)	11.1 (9.7, 12.7)	24.3 (22.3, 26.3)	
Education level	Low	42.4 (41.3, 43.4)	41.7 (39.3, 44.1)	41.1 (38.5, 43.7)	19.5 (17.6, 21.5)	48.1 (45.9, 50.3)	58.2 (56.1, 60.3)	<0.001
	Medium	22.3 (21.5, 23.0)	32.8 (30.7, 34.9)	33.8 (31.7, 35.9)	13.2 (11.5, 15.1)	23.0 (21.4, 24.6)	9.8 (8.9, 10.7)	
	High	35.3 (34.5, 36.2)	25.6 (23.7, 27.5)	25.1 (23.4, 26.8)	67.2 (64.8, 69.5)	28.9 (27.3, 30.6)	31.9 (30.1, 33.8)	
Nutrition knowledge	Not knowledgeable	38.0 (36.9, 38.9)	36.6 (34.4, 38.9)	34.2 (31.9, 36.6)	33.2 (30.9, 35.6)	48.5 (46.3, 50.6)	34.6 (32.3, 36.9)	<0.001
	Somewhat knowledgeable	42.7 (41.6, 43.7)	41.5 (39.2, 43.8)	44.4 (41.9, 46.8)	52.9 (50.4, 55.4)	35.5 (33.5, 37.6)	41.1 (38.7, 43.3)	
	Knowledgeable	19.4 (18.6, 20.2)	21.9 (20.0, 23.8)	21.4 (19.6, 23.3)	13.8 (12.2, 15.6)	16.0 (14.5, 17.6)	24.3 (22.4, 26.3)	
Income adequacy	Very difficult or difficult	30.7 (29.7, 31.6)	28 (25.9, 30.1)	28.4 (26.1, 30.7)	43.9 (41.4, 46.4)	25.4 (23.5, 27.3)	29.5 (27.3, 31.7)	<0.001
	Neither easy nor difficult	36.4 (35.4, 37.5)	37.4 (35.2, 39.8)	36.6 (34.3, 39.0)	38.7 (36.3, 41.1)	36.0 (33.9, 38.1)	33.8 (31.5, 36.1)	
	Easy or very easy	32.9 (31.9, 33.8)	34.6 (32.4, 36.8)	35.0 (32.8, 37.2)	17.4 (15.7, 19.2)	38.6 (36.5, 40.7)	36.7 (34.5, 39.0)	

1 Data were weighted using survey weights. Sample weights were constructed using population estimates from the census in each country based on age group, gender, and region.

2 99% CI = 99% CIs.

3 Linear regression and Pearson χ2 tests were calculated to determine differences by sociodemographic characteristics and ethnicity.

International Food Policy Study.

### University setting

In university settings, sugary drinks and junk food were reported to be available for purchase by 67–85% of participants (Figure 1C), whereas fruits and vegetables and other healthy snacks were reported to be available for purchase by 56–73% of participants. Water was reported to be available for free by 32.8–51.4% of participants (Figure 1B).

Across countries, participants in Canada (72.4%) and the UK (67.4%), were less likely than those in Mexico (81.6%) to report that junk food was available for purchase (range of RRRs = 0.48–0.56) (Figure 1C). Those in Australia (78.1%) and the UK (74.0%) were less likely to report that sugary drinks were available for purchase in universities compared with participants in Mexico (84.9%) (range of RRRs: 0.45–0.47). There were no between-country differences in the availability of junk food or sugary drinks for free in this setting.

Regarding healthy foods, participants from all countries (61.0–67.0%) were more likely to report the availability of fruits and vegetables for purchase than Mexican participants (56.6%) (range of RRRs = 1.89–3.32). Those in the UK (65.3%) and the USA (67%) were more likely to report the availability of fruits and vegetables for purchase in the university setting than those in Australia (63.8%) (range of RRRs: 1.71–1.75) and Canada (61.0%) (range of RRRs: 1.70–1.75) (Figure 1C). The pattern of findings was the same for fruits and vegetables available for free, with the exception that there was no difference between Canada (20.1%), the USA (21.2%), and the UK (22.8%) (Figure 1B). Participants in Mexico (21.3%) were more likely to report that other healthy snacks were not available in this setting compared with the rest of the countries (12.9–16.0%) (range of RRRs: 2.11–2.91) (Figure 1A). Mexican participants (62.3%) were more likely to report that water was available for purchase in the university setting compared with Canadian participants (40.5%) (RRR: 2.54; 99% CI: 1.23–5.23) (Figure 1C). There were no between-country differences in the availability of water for free in the university setting.

### Workplace

Across countries, around 50% of participants reported that junk food and sugary drinks were available for purchase; meanwhile ∼30–40% perceived that fruits and vegetables and other healthy snacks were available for purchase (Figure 2C). Most reported that water was available for free (Figure 2B).

Some differences across countries were observed (Supplemental Table 3). Junk food was perceived as more available for purchase in the workplace in Mexico (69.9%) compared with the rest of the countries (45.6–53.5%) (range of RRRs: 1.57–2.79). Participants in the USA (53.5%) were more likely than those in Australia (50.8%), Canada (49.0%), and the UK (45.6%) to report that these foods were available for purchase (range of RRRs: 1.37–1.77); whereas those in Australia (RRR: 1.28, 99% CI: 1.06–1.56) reported higher availability than participants in the UK (Figure 2C). US participants (17.3%) were more likely to report the availability of junk food for free in the workplace compared with those in the rest of the countries (5.8–11.0%) (range of RRRs: 2.07–2.54) (Figure 2B). A similar pattern as the one observed for the availability of junk food for purchase was observed for sugary drinks for purchase. Participants in Australia (32.7%), Canada (34.9%), and the UK (37.4%) were more likely to report that sugary drinks were not available in the workplace than those in Mexico (17.3%) (range of RRRs:1.66–1.91) and the USA (26.6%) (range of RRRs: 1.76–2.02) (Figure 2A).

Regarding healthy foods, UK (40.2%) participants were more likely to report the availability of fruits and vegetables for purchase in the workplace than the rest of the countries (34.2–38.0%) (range of RRRs: 1.24–1.52). Fruits and vegetables were more available for free in this setting in Australia (20.2%) and the UK (18.5%) than in Canada (12.3%) (range of RRRs: 1.95–1.97) and Mexico (9.6%) (range of RRRs: 2.44–2.47); and in the USA compared with Canada (RRR: 1.56; 99% CI: 1.16–2.09) and Mexico (RRR: 1.96, 99% CI: 1.48–2.59) (Figure 2B). Mexican (47.1%) and US (45.6%) participants were more likely to report that other healthy snacks were available for purchase in the workplace than those in Australia (42.0%) (range of RRRs: 1.23–1.30) and Canada (41.4%) (range of RRRs: 1.27–1.34). US (15.0%) participants were more likely to report that other healthy snacks were available for free in the workplace compared with the rest of the countries (≈10%) (range of RRRs:1.55–1.75). Mexican (48.1%) participants were more likely to report that water was available for purchase in the workplace compared with the rest of the countries (25.8–32.5%) (range of RRR: 2.63–3.40). Participants in the UK (10.8%) and the USA (10.0%) were more likely than Mexican participants (5.4%) to report that water was not available in this setting (range of RRRs: 1.50–1.53) (Figure 2A).

### Community

Most participants (>80%) in all countries perceived most categories to be available for purchase in the community setting (Figure 3).

Small but significant differences in the perceived availability of foods for purchase in the community were observed across countries (Supplemental Table 2). Overall, UK (85.1%) participants were less likely than those from Australia (87.5%), Canada (88.8%), and the USA (88.8%) to report that junk food was available for purchase in their community (range of RRRs: 0.71–0.81). Participants in Mexico (87.7%) were less likely to report that sugary drinks were available for purchase in the community than those in Australia (90.3%), Canada (90.6%), and the UK (90.1%) (range of RRRs: 0.71–0.78).

Concerning healthy foods, UK (89.3%) and Australian (87.7%) participants were more likely to report fruit and vegetable availability for purchase than those in the rest of the countries (83.8–85.3%, range of RRRs: 1.16–1.61). Participants in all countries were more likely (85.8–86.3) to report the availability of other healthy snacks for purchase in the community compared with Mexico (74.7%) (range of RRRs: 2.04–2.13). Participants in the USA (92.3%), Mexico (93.0%), and Canada (92.3%) were more likely to report the availability of water for purchase in the community than those in the UK (90.0%) (range of RRRs: 1.26–1.47).

## Discussion

Perceived availability of food and beverages varied across countries and settings. In the community, healthy and unhealthy foods were perceived as highly available across countries; in university and workplace settings sugary drinks and junk food were highly perceived as available across countries, with lower availability for fruits and vegetables and other healthy foods. In addition, few participants reported that water was not available across settings and countries. These findings are in line with trends in the global demand and supply of food suggesting an increase of unhealthy foods which may have displaced healthier options (4). The fact that unhealthy foods are highly available is important since studies support a relation between perceived food availability and dietary intake and diet-related outcomes (56–60). This becomes even more relevant for spaces where people spend most of their time and have access to a significant part of the food they eat, such as university and workplace settings.

Overall, the results of this study are somewhat in line with an 11-country study comparing the implementation of recommended food environment policies (56). Among the countries included in that study, UK had the highest proportion of policies rated at “high” implementation, most policies were rated as “low” or “medium” implementation in Australia and Canada, whereas Mexico had the highest proportion of policies rated at “very low if any” implementation. In a broader sense, our results are also in line with the higher availability of food environment policies addressing the school, workplace, and university settings in the UK compared with the rest of the countries (Supplemental Table 1).

Our study showed that the UK had the highest availability of free fruits and vegetables and other healthy snacks, and the lowest availability of junk food at the university setting. Although no specific regulations targeted at higher education centers exist in the UK, the high perceived availability of healthy foods in these settings may be explained by different strategies aiming to improve the healthiness of foods offered by catering services, or spillover effects of existing standards for school food or programs aimed to increase the intake of fruit and vegetables in school settings (29, 30, 61). The USA also tended to have high perceived availability for purchase or for free fruits and vegetables and other healthy snacks in the university setting, which is in line with voluntary regulations for food and beverage sales in the campuses of some US universities. For example, the Healthier Campus Initiative through the Partnership for a Healthier America, aiming to make nutritious foods and opportunities for physical activity both accessible and built into the campus culture (18, 19). However, our results also indicate high availability of junk food and sugary beverages for free in these settings, which may be explained by the voluntary nature of existing regulations or give-away events. In contrast, Mexico had the highest perceived availability of unhealthy foods and beverages for purchase in universities. These findings may be explained by the fact that in Mexico no regulations exist for the promotion or expenditure of foods in these settings. Overall, the findings underscore the importance of implementing and enforcing mandatory programs aimed at providing healthy food environments in universities. Several examples exist on how to develop and implement policies in these settings to ensure access to healthier foods (19).

Results of our study also showed that the UK had the lowest perceived availability of junk food and sugary drinks in the workplace, which is in line with available regulations and guidelines to provide healthy foods and beverages at these settings (30, 61). Similarly, the USA had the highest reported availability of free healthy snacks in the workplace, which may be due to healthy food procurement policies requiring that the food in specific settings (e.g. school, work, or community) is healthy (62). However, junk food and sugary drinks were also highly available for free in US work settings which may also be explained by the voluntary nature of the initiatives and guidelines (20, 21). Junk food and sugary drinks were also highly available at Mexican workplaces, along with the low perceived availability of free healthy foods in this setting. In this country, no mandatory regulations exist regarding the procurement of healthy foods in this setting. The only available regulation addresses the nutritional standards for voluntary food assistance programs (i.e. meals, food stamps, or food baskets) for Mexican workers, which may not be offered by all employers (16). These results underscore the need for a better implementation of the existing food environment regulations in the workplace. Effective interventions to promote dietary changes may include increasing access to healthy food or reducing prices of healthy snacks in vending machines (63). Investments in healthy food environments in these settings have shown their potential to reduce healthcare costs as well as overall absenteeism (64).

Our results suggest that the community setting had the largest perceived availability of healthy and unhealthy foods across the studied settings. This finding is in line with studies documenting a high density of grocery stores and an increasing number of convenience stores and supermarkets in some of the countries studied (65, 66). However, variations across countries were observed for specific foods, which may be explained by the nature of implemented food policies. For example, Australia and Canada were the countries with the highest perceived availability of junk food and sugary drinks in this setting. Although both countries have implemented policies with potential impacts on the availability of foods and beverages at the community level (e.g. subsidies on healthy foods, strategies to increase access to healthy foods in remote or underserved communities) (8–11), to the authors’ knowledge no zoning or land-use regulations addressing the placing and access to healthy and unhealthy food outlets have been implemented in these countries. This may explain the observed high availability of unhealthy foods in these settings, in line with previous studies (67, 68). In contrast, the role of planning in promoting healthy communities has been recognized in policy documents and regulations in the UK, where according to our results a higher perceived availability of healthy foods was reported (34). One of the most significant differences across countries was the low perceived availability of fruits and vegetables and other healthy snacks in Mexican communities. Despite the fact that local production of fruits and vegetables is sufficient for the Mexican population, calls have been made to develop policies to improve access to healthy foods and guarantee their equitable distribution (69). Differences in the prevalence of different types of stores (e.g. public markets or small food stores compared with chain supermarkets or convenience stores), pedestrian access to these outlets, as well as differences in transportation modes across countries, may also help explain some of the above-mentioned differences, warranting further studies across different cultural settings (70, 71). However, results underscore the need for strategies aiming to increase the availability of healthy foods in the community setting as well as information campaigns to help people identify healthy foods and healthier retail food environments. Interventions focusing on the in-store food environment, placing fruits and vegetables at the end-aisle to make them more visible and appealing to the consumer, and implementing promotions like 2-for-1 sales for healthy food options, may also be desirable (35).

Findings of this study build on previous findings suggesting the high availability of unhealthy foods across settings (4) and contribute to filling the gap in the literature regarding perceived availability among countries. Future studies should explore other components of the food environment, such as food accessibility and affordability (3), a broader variety of countries of low- and middle-income, and specific questions related to individual policies. Further, results of this study should be interpreted within the context of several limitations. Participants were recruited using nonprobability-based sampling; therefore, findings do not provide nationally representative estimates. The instrument used to measure the food environment has not been validated against objective measures. Respondents were not provided with examples or definitions for the included food groups, making interpretations susceptible to subjectivity and cultural differences across countries. Adjusted models may have partially addressed this issue by considering covariates that could be related to such subjectivity (i.e. nutrition knowledge, age, ethnicity, education level, or income adequacy). Analyses did not consider the type of setting (public or private), which could have been useful to further investigate differences in the perceived availability of foods across settings. Finally, results do not allow identifying the level of difference in access these results represent (e.g. regularly available versus occasionally available).

In conclusion, across countries, there was high perceived availability of unhealthy foods in all settings, and in school and work settings they were more available than healthy foods. Some variability between countries was documented which may reflect differences in policies and regulations targeting the food environment, as well as their degree of enforcement. Our results underscore the need for the continuation and improvement of policy efforts to generate healthier food environments.

## Supplementary Material

nxac070_Supplemental_File

## Notes

This supplement was supported by funding from a Project Grant from the Canadian Institutes of Health Research (PJT-162167). The views expressed herein are solely the responsibility of the authors and do not necessarily represent the official views of the Canadian Institutes for Health Research or other sources of funding. Funding for the International Food Policy Study was provided by a Canadian Institutes of Health Research (CIHR) Project Grant, with additional support from an International Health Grant, the Public Health Agency of Canada (PHAC), and a CIHR – PHAC Applied Public Health Chair (DH). JA is supported by the Medical Research Council (grant number MC_UU_00006/7). Funders had no role in designing the study, collecting, analyzing, and interpreting the data, drafting the manuscript nor the decision to publish findings. The supporting sources had no involvement or restrictions regarding publication.

Author disclosures: AC-M works in a civil society organization funded by Bloomberg Philanthropies. CN was awarded the healthy food policy fellowship from Vital Strategies. DH has served as a paid expert witness on behalf of public health authorities in the legal challenge to San Francisco's health warning ordinance for sugar-sweetened beverages. All other authors report no conflicts of interest.

Publication costs for this supplement were defrayed in part by the payment of page charges. The opinions expressed in this publication are those of the authors and are not attributable to the sponsors or the publisher, Editor, or Editorial Board of *The Journal of Nutrition*.

Supplemental Tables 1–4 are available from the “Supplementary data” link in the online posting of the article and from the same link in the online table of contents at https://academic.oup.com/jn/.

## References

[1] Dai H , Alsalhe TA, Chalghaf N, Riccò M, Bragazzi NL, Wu J. The global burden of disease attributable to high body mass index in 195 countries and territories, 1990–2017: an analysis of the Global Burden of Disease Study. PLoS Med. 2020;17(7):e1003198. 32722671 10.1371/journal.pmed.1003198 PMC7386577

[2] Glanz K , Sallis JF, Saelens BE, Frank LD. Healthy nutrition environments: concepts and measures. Am J Health Promot. 2005;19(5):330–33. 15895534 10.4278/0890-1171-19.5.330

[3] Caspi CE , Sorensen G, Subramanian SV, Kawachi I. The local food environment and diet: a systematic review. Health Place. 2012;18(5):1172–87. 22717379 10.1016/j.healthplace.2012.05.006 PMC3684395

[4] Global Panel on Agriculture and Food Systems for Nutrition . 2016. Food Systems and Diets: Facing the Challenges of the 21st Century. London, UK. [Internet]. Available at: http://glopan.org/sites/default/files/ForesightReport.pdf.

[5] Swinburn B , Vandevijvere S, Kraak V, Sacks G, Snowdon W, Hawkes C et al. INFORMAS. Monitoring and benchmarking government policies and actions to improve the healthiness of food environments: a proposed Government Healthy Food Environment Policy Index. Obes Rev. 2013. 10.1111/obr.12073. 24074208

[6] Shi Y , Wang Q, Norman C, Allman-Farinelli M, Colagiuri S. It is time to make policy for healthier food environments in Australian universities. Nutrients. 2018;10(12):1909. 10.3390/nu10121909 PMC6316519 30518049

[7] Policies for Tackling Obesity and Creating Healthier Food Environments. Progress update. Australian governments. 2019. [Internet]. Available at: https://docs.wixstatic.com/ugd/2e3337_5d2fdb48e7114f2c8cd14e79cb194393.pdf.

[8] Lee AJ , Kane S, Ramsey R, Good E, Dick M. Healthy Food Environment Policy Index (Food-EPI) - Testing the Price and Affordability of Healthy and Current (Unhealthy) Diets and the Potential Impacts of Policy Change in Australia. BMC Public Health. 2016; Apr. [Internet]. DOI: 10.1186/s12889-016-2996-y. https://bmcpublichealth.biomedcentral.com/track/pdf/10.1186/s12889-016-2996-y.pdf. 10.1186/s12889-016-2996-y PMC4828857 27067642

[9] Australian Government . Department of Health. What we're Doing about Food and Nutrition,2021.[Internet]. Available at: https://www.health.gov.au/health-topics/food-and-nutrition/what-were-doing.

[10] Brimblecombe J , McMahon E, Ferguson M, De Silva K, Peeters A, Miles E et al. Effect of restricted retail merchandising of discretionary food and beverages on population diet: a pragmatic randomised controlled trial. The Lancet Planetary Health. 2020;4(10):e463–73. 33038320 10.1016/S2542-5196(20)30202-3

[11] Vanderlee L , Goorang S, Karbasy K, Vandevijvere S, L'Abbé MR. Policies to create healthier food environments in Canada: experts' evaluation and prioritized actions using the healthy food environment policy index (Food-EPI). Int J Environ Res Public Health. 2019;16(22):4473. 10.3390/ijerph16224473 PMC6888279 31739397

[12] Government of Canada . Healthy Eating at Work. Canada's Food Guide. 2021. [Internet]. Available at: https://food-guide.canada.ca/en/tips-for-healthy-eating/work/.

[13] Government of Canada . Healthy Eating at Work. Canadian Centre for Occupational Health and Safety. 2017. [Internet]. Available at: https://www.ccohs.ca/oshanswers/psychosocial/healthyeating.html.

[14] Government of Canada . Nutrition North Canada, 2021. [Internet]. Available at: https://www.nutritionnorthcanada.gc.ca/eng/1415385762263/1415385790537.

[15] Nieto C , Rodríguez E, Sánchez-Bazán K, Tolentino-Mayo L, Carriedo-Lutzenkirchen A, Vandevijvere S et al. The INFORMAS healthy food environment policy index (Food-EPI) in Mexico: an assessment of implementation gaps and priority recommendations. Obes Rev. 2019;20(S2):67–77. 30618143 10.1111/obr.12814

[16] Gobierno de México . Ley de Ayuda Alimentaria para Trabajadores. 2021. [Internet]. Available at: http://sil.gobernacion.gob.mx/Archivos/Documentos/2021/04/asun_4164841_20210407_1617829373.pdf.

[17] Partnership for a Healthier America . Healthier Campus Initiative: 2019 progress report. 2019. [Internet]. Available at: https://www.ahealthieramerica.org/progress-reports/2019/initiatives/healthier-campus-initiative.

[18] Food and Beverage Choices Policy . UC Berkeley University Health Services. 2020. [Internet]. Available at: .

[19] Rickrode-Fernandez Z , Kao J, Lesser MNR, Guess K. Implementation of a healthy food and beverage policy at a public university. J Nutr Educ Behav. 2021;53(10):891–9. 34373195 10.1016/j.jneb.2021.06.009

[20] Workplace Health Initiatives . The Center for Disease Control. 2017. [Internet]. Available at: https://www.cdc.gov/workplacehealthpromotion/initiatives/index.html.

[21] Centers for Disease Control and Prevention . Smart Food Choices: How to Implement Food Service Guidelines in Public Facilities. Atlanta, USA: 2018. [Internet]. Available at: https://www.cdc.gov/obesity/downloads/strategies/Smart-Food-Choices-508.pdf.

[22] Reinvestment Fund . FY 2020 Spending Package Boosts Farm Bill's Healthy Food Financing. 2019. [Internet]. Available at: https://www.reinvestment.com/news/2019/12/20/fy-2020-spending-package-boosts-farm-bills-healthy-food-financing/.

[23] Reinvestment Fund . America's Healthy Food Financing Initiative. US. 2020. [Internet]. Available at: https://www.investinginfood.com/.

[24] Story M , Kaphingst KM, Robinson-O'Brien R, Glanz K. Creating healthy food and eating environments: policy and environmental approaches. Annu Rev Public Health. 2008;29(1):253–72. 18031223 10.1146/annurev.publhealth.29.020907.090926

[25] Center for Nutrition Policy and Promotion (CNPP) . Food and Nutrition Service. 2014. US. [Internet]. Available at: https://www.fns.usda.gov/about-cnpp.

[26] Supplemental Nutrition Assistance Program (SNAP) . Food and Nutrition Service. 2020. US. [Internet]. Available at: https://www.fns.usda.gov/snap/supplemental-nutrition-assistance-program.

[27] Berkowitz SA , Curran N, Hoeffler S, Henderson R, Price A, Ng SW. Association of a fruit and vegetable subsidy program with food purchases by individuals with low income in the US. JAMA Network Open. 2021;4(8):e2120377. 34379125 10.1001/jamanetworkopen.2021.20377 PMC8358732

[28] Sprake EF , Russell JM, Cecil JE, Cooper RJ, Grabowski P, Pourshahidi LK et al. Dietary patterns of university students in the UK: a cross-sectional study. Nutrition Journal. 2018;17(1). DOI:10.1186/s12937-018-0398-y. 10.1186/s12937-018-0398-y PMC6172790 30290816

[29] Guidance School Food in England. 2021. [Internet]. Available at: https://www.gov.uk/government/publications/standards-for-school-food-in-england/school-food-in-england.

[30] Healthier Catering Commitment for London. 2022. UK. [Internet]. Available at: https://healthiercateringcommitment.co.uk/.

[31] The Workplace (Health, Safety and Welfare) Regulations 1992. [Internet]. Available at: https://www.hse.gov.uk/pubns/indg244.pdf.

[32] Government Buying Standards for food and catering services. UK 2021. [Internet]. Available at: https://www.gov.uk/government/publications/sustainable-procurement-the-gbs-for-food-and-catering-services/government-buying-standard-for-food-and-catering-services.

[33] Adams J , Halligan J, Burges Watson D, Ryan V, Penn L, Adamson AJ et al. The Change4Life convenience store programme to increase retail access to fresh fruit and vegetables: a mixed methods process evaluation. PLoS One. 2012; ;7(6):e39431. 22761795 10.1371/journal.pone.0039431 PMC3384642

[34] Local Government Association . Strategies for Encouraging Healthier ‘Out Of Home’ Food Provision aToolkit for Local Councils Working with Small Food Businesses. Public Health England. 2017. [Internet]. Available at: https://assets.publishing.service.gov.uk/government/uploads/system/uploads/attachment_data/file/832910/Encouraging_healthier_out_of_home_food_provision_toolkit_for_local_councils.pdf.

[35] Ejlerskov KT , Stead M, Adamson A, White M, Adams J. The nature of UK supermarkets' policies on checkout food and associations with healthfulness and type of food displayed: cross-sectional study. International Journal of Behavioral Nutrition and Physical Activity. 2018;15(1). DOI: 10.1186/s12966-018-0684-2. 10.1186/s12966-018-0684-2 PMC5996483 29891005

[36] National Governors’ Association. Food Standards Agency . Food Policy in Schools. A Strategic Policy Framework for Governing Bodies. 2007. [Internet]. Available at: https://www.london.gov.uk/what-we-do/health/healthy-schools-london/awards/sites/default/files/FoodPolicyGovernorGuidance.pdf.

[37] Pérez-Ferrer C , Barrientos-Gutierrez T, Rivera-Dommarco JA, Prado-Galbarro FJ, Jiménez-Aguilar A, Morales-Ruán C et al. Compliance with nutrition standards in Mexican schools and their effectiveness: a repeated cross-sectional study. BMC Public Health. 2018;18(1). DOI: 10.1186/s12889-018-6330-8. 10.1186/s12889-018-6330-8 PMC6307217 30591040

[38] Ministerio de Salud . Ley de Alimentos—Medidas para establecimientos educacionales. Chile. 2016. [Internet]. Available at: https://www.minsal.cl/ley-de-alimentos-medidas-para-establecimientos-educacionales/.

[39] Sánchez BN , Sanchez-Vaznaugh EV, Uscilka A, Baek J, Zhang L. Differential associations between the food environment near schools and childhood overweight across race/ethnicity, gender, and grade. Am J Epidemiol. 2012;175(12):1284–93. 22510276 10.1093/aje/kwr454 PMC3372311

[40] UC Berkeley University Health Services . Food and Beverage Choices Policy. UC Berkeley University Health Services. 2020. [Internet]. Available at: https://campuspol.berkeley.edu/policies/foodbeverage.pdf.

[41] Roy R , Kelly B, Rangan A, Allman-Farinelli M. Food environment interventions to improve the dietary behavior of young adults in tertiary education settings: a systematic literature review. Journal of the Academy of Nutrition and Dietetics. 2015;115(10):1647–1681.e1. 26271691 10.1016/j.jand.2015.06.380

[42] Food Service Guidelines Federal Workgroup . Food Service Guidelines for Federal Facilities. Washington, DC: U.S. Department of Health and Human Services; 2017. [Internet]. Available at: https://www.cdc.gov/obesity/downloads/guidelines_for_federal_concessions_and_vending_operations.pdf.

[43] Food-based Dietary Guidelines - United Kingdom . Food and Agriculture Organization of the United Nations. 2016. [Internet]. Available at: http://www.fao.org/nutrition/education/food-dietary-guidelines/regions/countries/united-kingdom/en/.

[44] Victoria State Government . Healthy Choices. 2020. [Internet]. Available at: https://www2.health.vic.gov.au/public-health/preventive-health/nutrition/healthy-choices-for-retail-outlets-vending-machines-catering.

[45] UK Government. Guidance . Sustainable procurement: the GBS for food and catering services. 2014. [Internet]. Available at: https://www.gov.uk/government/publications/sustainable-procurement-the-gbs-for-food-and-catering-services.

[46] Mayo ML , Pitts SB, Chriqui JF. Associations between county and municipality zoning ordinances and access to fruit and vegetable outlets in rural North Carolina, 2012. Preventing Chronic Disease. 2013; Dec 5;10. DOI: 10.5888/pcd10.130196. 10.5888/pcd10.130196 PMC3854873 24309091

[47] Brown H , Kirkman S, Albani V, Goffe L, Akhter N, Hollingsworth B et al. The impact of school exclusion zone planning guidance on the number and type of food outlets in an English local authority: a longitudinal analysis. Health Place. 2021;70:102600. 34118573 10.1016/j.healthplace.2021.102600 PMC8361782

[48] Lytle LA . Measuring the food environment: state of the science. Am J Prev Med. 2009;36(4):S134–44. 19285204 10.1016/j.amepre.2009.01.018 PMC2716804

[49] Turner C , Aggarwal A, Walls H, Herforth A, Drewnowski A, Coates J et al. Concepts and critical perspectives for food environment research: a global framework with implications for action in low- and middle-income countries. Global Food Security. 2018;18:93–101.

[50] Hammond D , White CM, Rynard VL, Vanderlee L. International Food Policy Study: Technical Report - 2019 Survey (Wave 3). University of Waterloo. December 2021. Available at: http://foodpolicystudy.com/methods/.

[51] Centers for Disease Control and Prevention . Behavioral Risk Factor Surveillance System, 2016. Available at: https://www.cdc.gov/brfss/annual_data/annual_2016.html.

[52] Centers for Disease Control and Prevention National Health and Nutrition Examination Survey. 2016. Available at: https://wwwn.cdc.gov/nchs/nhanes/continuousnhanes/default.aspx?BeginYear=2015.

[53] Statistics Canada . Canadian Community Health Survey - Annual Component (CCHS), 2013. Available at: https://www23.statcan.gc.ca/imdb/p2SV.pl?Function=getSurvey&Id=144170.

[54] Australia Bureau of Statistics . Census of Population and Housing, 2016. Available at: https://www.abs.gov.au/ausstats/abs@.nsf/lookup/by%20subject/1001.0~2016-17~main%20features~the%202016%20census%20of%20population%20and%20housing~10009.

[55] INEGI . Censo de Población y Vivienda 2010. Mexico. Consulta interactiva de datos, 2010. Available at: https://www.inegi.org.mx/programas/ccpv/2010/.

[56] Vandevijvere S , Barquera S, Caceres G, Corvalan C, Karupaiah T, Kroker-Lobos MF et al. An 11-country study to benchmark the implementation of recommended nutrition policies by national governments using the Healthy Food Environment Policy Index, 2015–2018. Obes Rev. 2019;20(S2):57–66. 30609260 10.1111/obr.12819

[57] Hayes JF , Balantekin KN, Conlon RPK, Brown ML, Stein RI, Welch RR et al. Home and neighborhood built environment features in family-based treatment for childhood obesity. Pediatric obesity. 2019;14(3):e12477. 30378768 10.1111/ijpo.12477 PMC6379099

[58] Bekelman T , Santamaría-Ulloa C, Dufour. Perceptions of food availability and self-reported dietary intake in urban Costa Rican women: a pilot study. Población y Salud en Mesoamérica. 2016;13(2). DOI: 10.15517/psm.v13i2.22164.

[59] Maddock J . The relationship between obesity and the prevalence of fast food restaurants: state-level analysis. Am J Health Promot. 2004;19(2):137–43. 15559714 10.4278/0890-1171-19.2.137

[60] Sturm R , Datar A. Body mass index in elementary school children, metropolitan area food prices and food outlet density. Public Health. 2005;119(12):1059–68. 16140349 10.1016/j.puhe.2005.05.007

[61] Workplace health, safety and welfare a short guide for managers. Health and Safety Executive. UK. 1992. [Internet]. Available at: https://www.hse.gov.uk/pubns/indg244.pdf.

[62] Niebylski ML , Lu T, Campbell NRC, Arcand J, Schermel A, Hua D et al. Healthy food procurement policies and their impact. Int J Environ Res Public Health. 2014;11(3):2608–27. 24595213 10.3390/ijerph110302608 PMC3986994

[63] Quintiliani L , Poulsen S, Sorensen G. Healthy eating strategies in the workplace. International Journal of Workplace Health Management. 2010;3(3):182–96. 23935706 10.1108/17538351011078929 PMC3737584

[64] Grimani A , Aboagye E, Kwak L. The effectiveness of workplace nutrition and physical activity interventions in improving productivity, work performance and workability: a systematic review. BMC Public Health. 2019;19(1):1676. 31830955 10.1186/s12889-019-8033-1 PMC6909496

[65] Pérez-Ferrer C , Auchincloss AH, Barrientos-Gutierrez T, Colchero MA, de Oliveira Cardoso L et al. Longitudinal changes in the retail food environment in Mexico and their association with diabetes. Health Place. 2020;66:102461. 33039800 10.1016/j.healthplace.2020.102461 PMC7705211

[66] Hilmers A , Hilmers DC, Dave J. Neighborhood disparities in access to healthy foods and their effects on environmental justice. Am J Public Health. 2012;102(9):1644–54. 22813465 10.2105/AJPH.2012.300865 PMC3482049

[67] Cervigni E , Renton M, Haslam MF, HicklingS OD. Describing and mapping diversity and accessibility of the urban food environment with open data and tools. Appl Geogr. 2020;125:102352.

[68] Vanderlee L , Goorang S, Karbasy K, Schermel A, L'Abbe´M. Creating Healthier Food Environments in Canada: Current Policies and Priority Actions. 2017. [Internet]. Available at: http://labbelab.utoronto.ca/wp-content/uploads/2017/12/FoodEPI_ExecSum-WEB-FINAL.pdf.

[69] Valencia-Valero RG , Ortiz-Hernández L. [Food availability according to food security-insecurity among Mexican households]. Salud Publica Mex. 2014;; 56(2):154–64. 25014422

[70] Díez J , Bilal U, Cebrecos A, Buczynski A, Lawrence RS, Glass T et al. Understanding differences in the local food environment across countries: a case study in Madrid (Spain) and Baltimore (USA). Prev Med. 2016;89:237–44. 27311334 10.1016/j.ypmed.2016.06.013

[71] Pettinger C , Holdsworth M, Gerber M. ‘All under one roof?’ differences in food availability and shopping patterns in Southern France and Central England. Eur J Public Health. 2008;18(2):109–14. 17575310 10.1093/eurpub/ckm037

