# Supplementary material for: Perceived Availability of Healthy and Unhealthy Foods in the Community, Work, and Higher Education Settings across Five Countries: Findings from the International Food Policy Study 2018
Source: J Nutr. 2022 May 11;152(Suppl 1):47S–56S. doi: 10.1093/jn/nxac070 (PMC9188857; doi:10.1093/jn/nxac070)
Supplement: nxac070_Supplemental_File [file nxac070_supplemental_file.docx]

**Perceived availability of healthy and unhealthy foods in the community, work, and higher education settings across five countries: Findings from the International Food Policy Study 2018.**

**First author: Alejandra Contreras-Manzano**

**Online Supplementary Material**

**Supplemental Table 1.** **Examples of public policies related to food availability in the university, work and community setting in Mexico, Australia, United Kingdom (UK), United States (US), and Canada.**

| **Country** | **University setting** | **Work setting** | **Community setting** |
| --- | --- | --- | --- |
| Australia | Specific food policies in the university setting are not available; the need to make healthier choices more accessible to young adults has been highlighted.(5)    The Victoria territory improved the supply and promotion of healthy food and drinks within public settings, by increasing the scale and uptake of the Healthy Choices guidelines and aligned policies within early childhood settings, schools, health care services, sport and recreation facilities, parks, and universities.(6)  In the Australian Capital Territory, there is regular monitoring of school canteen menus, demonstrating high levels of compliance with the National Healthy School Canteen Guidelines.  New South Wales implemented comprehensive food provision policies in schools and health facilities, including detailed auditing and compliance monitoring.  There is also the implementation of the Tasmanian School Canteen Accreditation program, leading to a rapid increase in the number of schools accredited and participating in the program. | Local improvements in the availability of unhealthy foods have been documented for some work settings. For example, Western Australia banned the sale of unhealthy food and drinks in hospitals and other state-owned institutions.(6) | There are several local and national policies that have potential impacts on the availability of foods and beverages (7) in the community setting. For example; a voluntary labelling system (the Health Star Rating),(8) exemption of ‘healthy’ foods from goods and service tax or value-added tax e.g. fruit, vegetables, bread, fresh meat, milk, and eggs.(7) Subsidies on healthy foods, such as agricultural and transport subsidies, retail price reductions, or voucher systems, are targeted to high-risk groups.  In the Northern Territory there is ongoing support for remote communities/stores to improve the availability of healthy foods and limit the availability/sales of unhealthy foods.  In Tasmania there are funding grants for community access to local, healthy foods.(6)  The National Healthy Food Partnership is a collaboration between the government, the food industry, and public health sector to make healthier food choices easier and more accessible.(6). The program Eat for Health gives advice and resources to help people make healthy food choices.  Other healthy food intervention implemented in remote communities in Australia has been reported previously, like The Healthy Stores 2020 strategy with aimed to restrict merchandising of unhealthy foods and beverages, this community intervention resulted in a reduction in sales of free sugar of 2.8% on average in three months.(9) |
| Canada | There is reasonably high uptake of school nutrition policies or nutritional standards in schools in Canada, although implementation and effectiveness of these policies differs between provinces, school boards and schools. (10) There are no specific government policies in university settings. | Voluntary guidelines for making healthier choices more available in the work setting have been established for Canada.(11, 12)  Reports indicate low implementation of private company nutrition policies (38%), and moderate implementation of public sector nutrition policies (48%).(10) | A healthy food intervention implemented in remote communities in Canada is the Nutrition North program, which supports in-store availability and promotion of healthy foods in remote northern communities.(13)  Canada has low or no taxes for ‘basic groceries’ in Canada. There are no national of provincial policies related to improving healthy food availability in food stores or restaurants, or restrictions in density or placement of healthy or unhealthy food outlets. (10) |
| Mexico | Regulations to provide free drinking water in schools and universities, as well for the expenditure of healthy foods in basic education centers are implemented in the country, however their implementation is low.  No regulations exist to restrict the promotion or expenditure of unhealthy foods in university campuses and there is low government support and training systems for food suppliers in schools and public education institutions.(14) | The Food Assistance Law for Workers outlines the nutritional requirements for all food assistance (meals, food baskets or food stamps) provided to workers. The provision of food assistance is voluntary.(15)  There is low implementation of government support and training systems for food suppliers, provision and promotion of healthy foods in public institutions and work settings.(14) | Although several public policies related to the food environment have been put in place in the country (e.g., soda tax, front-of-pack labelling), none of them target the availability, accessibility or affordability of healthy and unhealthy foods at the community setting.(14) |
| US | There are some local and mandatory regulations related to food availability in the University setting, for example; “The Healthier Campus Initiative through the Partnership for a Healthier America”,(16) or “Food and Beverage Choices Policy. UC Berkeley”(17) and others for public universities.(18) | The Center for Disease Control provides national leadership and support for employee health, safety, and well-being in the worksite setting. CDC’s approach includes science-based and scalable interventions and prevention strategies targeting the employers that are most affected by the costs of poor employee health.(19, 20) | The America’s Healthy Food Financing Initiative aims to improve access to healthy food and expand economic opportunity in low economic settings by providing grants to states to support and attract healthier retail outlets and ‘food enterprises’ to areas that are currently under-served.(21, 22)  There exist some voluntary and local policies to create healthy food and eating environments at the community level,(23) also there are some national programs for nutrition promotion (24) and other that provides nutrition benefits for low-income families so they can purchase healthy food.(25, 26) |
| UK | There is lack of national policies directed to improve nutrition quality of foods and beverages at the university setting and the need to target interventions towards students who consume poor diets and practice unfavorable lifestyle behaviors has been recognized.(27)  Legislations to food provided within schools in England are in place since 2015.(28) Food served in some schools and academies must meet the school food standards so that children have healthy, balanced diets. Junk food is not allowed in these settings.  Several school caterers have signed up to the Healthier Catering Commitment, a scheme committed to reducing the levels of saturated fat, salt and sugar in foods sold across London.(29) | The Workplace (Health, Safety and Welfare) Regulations require all workplaces provide an adequate supply of high-quality drinking water.(30)  Several workplace caterers have signed up to the Healthier Catering Commitment, a scheme committed to reducing the levels of saturated fat, salt and sugar in foods sold across London.(29)  Mandatory buying standards for the National Health Service trusts (food and catering services) to help increase the overall performance, delivery and quality of hospital food. For example; a portion of fruit shall be sold at a lower price than a portion of hot or cold dessert, or no more than 10% beverages provided can be sugar sweetened beverages, or tap water is visible and freely available and such provision is promoted.(31) | The “Change4Life Convenience Store Programme”, aimed to increase retail access to fresh fruit and vegetables, did not have a potential impact on consumers’ consumption.(32)  Several guidelines exist that advocate for healthy communities and healthy food environments. The National Planning Policy Framework recognizes the role of planning in promoting the delivery of healthy communities.(33) The Guidance on healthy and safe communities specifically mentions the range of issues that could be considered through the plan-making and decision-making processes, including access to healthier food as part of the opportunities to promote healthy lifestyles. Regulations for granting planning permission to new hot food takeaways are also in place in some local councils.(33) Local authorities have also implemented award schemes to promote the sale of healthy foods, as well as strategies to increase their fruit and vegetable sales by linking with the NHS’s Healthy Start Vouchers scheme (vouchers are supplied to low income families for the purchase of fresh fruit and vegetables from local stores and markets).  In the UK most supermarkets now have self-policed policies on less healthy food at checkouts and there are proposals in place to ban placement of less healthy foods at end of aisle, checkout, store entrance which may led to changes in advance of implementation.(34) |

**Supplemental Table 2.** **Country comparisons of perceived availability of foods and drinks in the university setting (n=3,253)**

|  | **Junk food** | | | | **Sugary drinks** | | | | **Fruits and vegetables** | | | | **Other healthy snacks** | | | | **Water** | | | |
| --- | --- | --- | --- | --- | --- | --- | --- | --- | --- | --- | --- | --- | --- | --- | --- | --- | --- | --- | --- | --- |
| **Not available (ref)** | **Available for purchase** | | **Available for free** | | **Available for purchase** | | **Available for free** | | **Available for purchase** | | **Available for free** | | **Available for purchase** | | **Available for free** | | **Available for purchase** | | **Available for free** | |
| **Country** | **RRR^1^ (99% CI)** | **p value** | **RRR^1^**  **(99%**  **CI)** | **p value** | **RRR^1^ (99% CI)** | **p value** | **RRR^1^**  **(99% CI)** | **p value** | **RRR^1^**  **(99% CI)** | **p**  **value** | **RRR^1^**  **(99% CI)** | **p value** | **RRR^1^**  **(99% CI)** | **p value** | **RRR^1^**  **(99% CI)** | **p value** | **RRR^1^ (99% CI)** | **p value** | **RRR^1^ (99% CI)** | **p value** |
| Australia (ref) vs. Canada | 0.79  (0.45, 1.40) | 0.299 | 0.71  (0.34, 1.50) | 0.247 | 1.28  (0.66, 2.49) | 0.328 | 1.26  (0.55, 2.87) | 0.468 | 1.00  (0.60, 1.65) | 0.995 | 1.28  (0.66, 2.47) | 0.330 | 0.83  (0.48, 1.43) | 0.391 | 0.93  (0.44, 1.95) | 0.823 | 0.76  (0.34, 1.69) | 0.386 | 0.98  (0.44, 2.18) | 0.962 |
| Australia (ref) vs.Mexico | 1.41  (0.81, 2.44) | 0.103 | 0.79  (0.39, 1.58) | 0.395 | **2.20**  **(1.19, 4.09)** | **0.001** | 1.85  (0.85, 4.02) | 0.038 | **0.52**  **(0.35, 0.78)** | **<0.001** | **0.37**  **(0.21, 0.64)** | **<0.001** | **0.61**  **(0.39, 0.96)** | **0.006** | **0.44**  **(0.23, 0.82)** | **0.001** | 1.94  (0.97, 3.89) | 0.013 | 1.04  (0.51, 2.10) | 0.882 |
| Australia (ref) vs. US | 0.90  (0.50, 1.62) | 0.661 | 1.12  (0.55, 2.27) | 0.668 | 1.16  (0.60, 2.24) | 0.554 | 1.92  (0.87, 4.23) | 0.032 | **1.75**  **(1.02, 3.00)** | **0.007** | **2.15**  **(1.10, 4.17)** | **0.003** | 1.04  (0.58, 1.84) | 0.851 | 1.29  (0.63, 2.66) | 0.350 | 1.25  (0.54, 2.87) | 0.478 | 0.99  (0.43, 2.28) | 0.982 |
| Australia (ref) vs. UK | 0.67  (0.40, 1.14) | 0.057 | 0.84  (0.43, 1.63) | 0.510 | 1.05  (0.58, 1.89) | 0.831 | 1.66  (0.79, 3.50) | 0.075 | **1.71**  **(1.02, 2.84)** | **0.007** | **2.32**  **(1.24, 4.33)** | **0.001** | 0.94  (0.56, 1.58) | 0.782 | 1.27  (0.65, 2.48) | 0.352 | 1.27  (0.58, 2.77) | 0.427 | 1.42  (0.65, 3.09) | 0.239 |
| Canada (ref) vs. Mexico | **1.77**  **(1.00, 3.13)** | **0.009** | 1.10  (0.52, 2.35) | 0.723 | 1.71  (0.87, 3.35) | 0.370 | 1.47  (0.64, 3.37) | 0.227 | **0.52**  **(0.33, 0.82)<** | **0.001** | **0.29**  **(0.15, 0.52)** | **<0.001** | 0.73  (0.46, 1.17) | 0.093 | **0.47**  **(0.24, 0.92)** | **0.004** | **2.54**  **(1.23, 5.23)** | **0.001** | 1.05  (0.51, 2.18) | 0.846 |
| Canada (ref) vs. US | 1.13  (0.62, 2.08) | 0.586 | 1.56  (0.73, 3.34) | 0.127 | 0.90  (0.46, 1.77) | 0.702 | 1.52  (0.68, 3.39) | 0.172 | **1.75**  **(0.99, 3.09)** | **0.001** | 1.67  (0.84, 3.32) | 0.051 | 1.24  (0.70, 2.21) | 0.319 | 1.38  (0.66, 2.89) | 0.256 | 1.64  (0.76, 3.53) | 0.097 | 1.00  (0.46, 2.17) | 0.980 |
| Canada (ref) vs. UK | 0.85  (0.50, 1.44) | 0.440 | 1.17  (0.58, 2.37) | 0.549 | 0.81  (0.43, 1.53) | 0.406 | 1.32  (0.60, 2.89) | 0.355 | **1.70**  **(0.99, 2.93)** | **0.001** | 1.80  (0.94, 3.46) | 0.019 | 1.13  (0.66, 1.91) | 0.542 | 1.35  (0.67, 2.71) | 0.255 | 1.66  (0.78, 3.52) | 0.082 | 1.44  (0.68, 3.04) | 0.202 |
| Mexico (ref) vs. US | 0.63  (0.36, 1.13) | 0.046 | 1.41  (0.69, 2.85) | 0.206 | **0.52**  **(0.27, 1.02)** | **0.013** | 1.03  (0.47, 2.26) | 0.908 | **3.32**  **(2.08, 5.30)<** | **0.001** | **5.77**  **(3.24, 10.27)** | **<0.001** | **1.69**  **(1.04, 2.73)** | **0.005** | **2.91**  **(1.55, 5.47)** | **<0.001** | 0.64  (0.30, 1.34) | 0.123 | 0.95  (0.45, 2.00) | 0.870 |
| Mexico (ref) vs. UK | **0.48**  **(0.29, 0.78)** | **<0.001** | 1.06  (0.55, 2.01) | 0.810 | **0.47**  **(0.26, 0.85)** | **0.001** | 0.89  (0.43, 1.83) | 0.699 | **3.23**  **(2.09, 4.99)<** | **0.001** | **6.22**  **(3.62, 10.68)** | **<0.001** | **1.53**  **(1.00, 2.35)** | **0.010** | **2.86**  **(1.61, 5.08)** | **<0.001** | 0.65  (0.32, 1.31) | 0.118 | 1.36  (0.67, 2.77) | 0.250 |
| US (ref) vs. UK | 0.75  (0.42, 1.31) | 0.190 | 0.75  (0.38, 1.47) | 0.275 | 0.90  (0.48, 1.68) | 0.672 | 0.86  (0.41, 1.80) | 0.617 | 0.97  (0.55, 1.71) | 0.905 | 1.07  (0.56, 2.07) | 0.767 | 0.90  (0.52, 1.57) | 0.648 | 0.98  (0.49, 1.93) | 0.941 | 1.01  (0.47, 2.16) | 0.966 | 1.43  (0.67, 3.06) | 0.219 |

**^1^RRR and 95% CI derived from multinomial logistic regression models adjusted for age, sex, race or ethnicity, education level, income adequacy and nutritional knowledge and weighted for post-stratification sampling weights. Reference country presented first.**

**Supplemental Table 3. Country comparisons of perceived availability of foods and drinks in the workplace (n=11,233)**

|  | **Junk food** | | | | **Sugary drinks** | | | | **Fruits and vegetables** | | | | **Other healthy snacks** | | | | **Water** | | | |
| --- | --- | --- | --- | --- | --- | --- | --- | --- | --- | --- | --- | --- | --- | --- | --- | --- | --- | --- | --- | --- |
| **Not available (ref)** | Available for purchase | | Available for free | | Available for purchase | | Available for free | | Available for purchase | | Available for free | | Available for purchase | | Available for free | | Available for purchase | | Available for free | |
| **Country** | **RRR^1^ (99% CI)** | **p value** | **RRR^1^ (99% CI)** | **p value** | **RRR^1^ (99% CI)** | **p value** | **RRR^1^ (99% CI)** | **p value** | **RRR^1^ (99% CI)** | **p value** | **RRR^1^ (99% CI)** | **p value** | **RRR^1^ (99% CI)** | **p value** | **RRR^1^ (99% CI)** | **p value** | **RRR^1^ (99% CI)** | **p value** | **RRR^1^ (99% CI)** | **p value** |
| Australia (ref) vs. Canada | 0.92  (0.75, 1.12) | 0.292 | 0.95  (0.69, 1.32) | 0.737 | 0.88  (0.72, 1.08) | 0.128 | 1.02  (0.73, 1.43) | 0.861 | 0.81  (0.65, 1.00) | 0.014 | **0.50**  ( **0.38, 0.67)** | **<0.001** | 0.96  (0.79, 1.18) | 0.696 | 0.97  (0.70, 1.33) | 0.816 | 0.79  (0.60, 1.04) | 0.097 | 0.90  (0.70, 1.16) | 0.426 |
| Australia (ref) vs.Mexico | **2.17**  (**1.75, 2.68)** | **<0.001** | 0.84  (0.58, 1.20) | 0.217 | **2.43**  (**1.93, 3.06)** | **<0.001** | **1.70**  (**1.19, 2.42)** | **<0.001** | 0.92  (0.74, 1.14) | 0.335 | **0.40**  (**0.30, 0.53)** | **<0.001** | **1.23**  (**1.01, 1.51)** | **0.006** | 1.04  (0.75, 1.44) | 0.731 | **2.62**  (**1.94, 3.54)** | **<0.001** | 1.15  (0.86, 1.53) | 0.334 |
| Australia (ref) vs. US | **1.37**  (**1.11, 1.71)** | **<0.001** | **2.06**  ( **1.49, 2.85)** | **<0.001** | **1.25**  (**1.00, 1.56)** | **0.008** | **1.80**  (**1.28, 2.52)** | **<0.001** | 0.96  (0.77, 1.20) | 0.659 | 0.79  (0.60, 1.03) | 0.025 | **1.30**  (**1.05, 1.61)** | **0.001** | **1.70**  (**1.24, 2.32)** | **<0.001** | 0.95  (0.71, 1.27) | 0.769 | 0.76  (0.58, 1.00) | 0.054 |
| Australia (ref) vs. UK | **0.77**  (**0.64, 0.94)** | **0.001** | 0.81  (0.59, 1.11) | 0.093 | **0.79**  (**0.65, 0.97)** | **0.004** | 0.88  (0.63, 1.24) | 0.364 | **1.23**  (**1.00, 1.52)** | **0.008** | 0.98  (0.76, 1.27) | 0.916 | 1.13  (0.93, 1.38) | 0.091 | 1.10  (0.80, 1.50) | 0.426 | 0.77  (0.59, 1.00) | 0.058 | 0.75  (0.58, 0.96) | 0.023 |
| Canada (ref) vs. Mexico | **2.36**  (**1.90, 2.91)** | **<0.001** | 0.87  (0.61, 1.26) | 0.361 | **2.75**  (**2.18, 3.47)** | **<0.001** | **1.66**  (**1.17, 2.34)** | **<0.001** | 1.13  (0.92, 1.39) | 0.115 | 0.79  (0.58, 1.08) | 0.056 | **1.27**  (**1.04, 1.56)** | **0.002** | 1.07  (0.77, 1.48) | 0.566 | **3.31**  (**2.49, 4.39)** | **<0.001** | 1.27  (0.97, 1.67) | 0.079 |
| Canada (ref) vs. US | **1.49**  (**1.20, 1.85)** | **<0.001** | **2.15**  (**1.56, 2.97)** | **<0.001** | **1.41**  (**1.13, 1.76)** | **<0.001** | **1.76**  (**1.27, 2.43)** | **<0.001** | 1.18  (0.95, 1.46) | 0.046 | **1.56**  (**1.16, 2.09)** | **<0.001** | **1.34**  (**1.09, 1.65)** | **<0.001** | **1.75**  (**1.28, 2.39)** | **<0.001** | 1.20  (0.91, 1.58) | 0.178 | 0.84  (0.65, 1.09) | 0.210 |
| Canada (ref) vs. UK | 0.84  (0.69, 1.02) | 0.028 | 0.84  (0.61, 1.16) | 0.185 | 0.90  (0.73, 1.10) | 0.194 | 0.86  (0.62, 1.20) | 0.268 | **1.52**  (**1.23, 1.86)** | **<0.001** | **1.95**  (**1.47, 2.58)** | **<0.001** | 1.17  (0.96, 1.42) | 0.036 | 1.13  (0.82, 1.54) | 0.034 | 0.97  (0.75, 1.25) | 0.178 | 0.83  (0.65, 1.05) | 0.124 |
| Mexico (ref) vs. US | **0.63**  (**0.51, 0.79)** | **<0.001** | **2.45**  (**1.74, 3.44)** | **<0.001** | **0.51**  (**0.40, 0.64)** | **<0.001** | 1.05  (0.76, 1.47) | 0.653 | 1.04  (0.84, 1.27) | 0.605 | **1.95**  (**1.48, 2.58)** | **<0.001** | 1.05  (0.86, 1.28) | 0.510 | **1.63**  (**1.21, 2.19)** | **<0.001** | **0.36**  (**0.27, 0.48)** | **<0.001** | **0.66**  (**0.50, 0.87)** | **0.004** |
| Mexico (ref) vs. UK | **0.35**  (**0.29, 0.43)** | **<0.001** | 0.96  (0.68, 1.35) | 0.783 | **0.32**  (**0.26, 0.40)** | **<0.001** | **0.52**  (**0.37, 0.72)** | **<0.001** | **1.34**  (**1.10, 1.62)** | **<0.001** | **2.44**  (**1.87, 3.20)** | **<0.001** | 0.91  (0.76, 1.10) | 0.239 | 1.05  (0.77, 1.42) | 0.654 | **0.29**  (**0.22, 0.38)** | **<0.001** | **0.65**  (**0.50, 0.84)** | **0.001** |
| US (ref) vs. UK | **0.56**  (**0.45, 0.69)** | **<0.001** | **0.39**  (**0.28, 0.53)** | **<0.001** | **0.63**  (**0.51, 0.79)** | **<0.001** | **0.49**  (**0.35, 0.68)** | **<0.001** | **1.28**  (**1.04, 1.59)** | **0.002** | 1.25  (0.96, 1.62) | 0.028 | 0.87  (0.71, 1.06) | 0.085 | **0.64**  (**0.47, 0.87)** | **<0.001** | 0.80  (0.61, 1.05) | 0.122 | 0.98  (0.75, 1.26) | 0.891 |

**^1^RRR and 99% CI derived from multinomial logistic regression models adjusted for age, sex, race or ethnicity, education level, income adequacy and nutritional knowledge and weighted for post-stratification sampling weights. Reference country presented first.**

**Supplemental Table 4.** **Country comparisons of perceived availability of foods and drinks for purchase in the community (n=21,369)**

|  | **Junk food** | | **Sugary drinks** | | **Fruits and vegetables** | | **Other healthy snacks** | | **Water** | |
| --- | --- | --- | --- | --- | --- | --- | --- | --- | --- | --- |
| **Available for purchase.** | **RRR^1^**  **(99% CI)** | **p**  **value** | **RRR^1^**  **(99% CI)** | **p**  **value** | **RRR^1^**  **(99% CI)** | **p**  **value** | **RRR^1^**  **(99% CI)** | **p**  **value** | **RRR^1^**  **(99% CI)** | **p**  **value** |
| Australia (ref) vs. Canada | 1.13  (0.91, 1.40) | 0.120 | 1.03  (0.82, 1.30) | 0.696 | **0.81**  **(0.66, 0.99)** | **0.007** | 0.96  (0.79, 1.17) | 0.650 | 1.06  (0.82, 1.36) | 0.527 |
| Australia (ref) vs.Mexico | 0.92  (0.74, 1.14) | 0.336 | **0.77**  **(0.60, 0.97)** | **0.005** | **0.72**  **(0.58, 0.89)** | **<0.001** | **0.47**  **(0.39, 0.57)** | **<0.001** | 1.17  (0.88, 1.54) | 0.142 |
| Australia (ref) vs. US | 1.14  (0.92, 1.41) | 0.113 | 0.87  (0.69, 1.09) | 0.123 | **0.72**  **(0.59, 0.88)** | **<0.001** | 0.97  (0.79, 1.19) | 0.726 | 1.05  (0.81, 1.35) | 0.612 |
| Australia (ref) vs. UK | **0.81**  **(0.67, 0.98)** | **0.005** | 0.98  (0.79, 1.21) | 0.813 | 1.16  (0.95, 1.41) | 0.046 | 1.01  (0.83, 1.22) | 0.876 | 0.79  (0.63, 0.99) | 0.007 |
| Canada (ref) vs. Mexico | 0.81  (0.64, 1.01) | 0.017 | **0.74**  **(0.58, 0.94)** | **0.001** | 0.89  (0.72, 1.09) | 0.149 | **0.49**  **(0.40, 0.59)** | **<0.001** | 1.10  (0.82, 1.46) | 0.379 |
| Canada (ref) vs. US | 1.00  (0.80, 1.25) | 0.959 | 0.84  (0.66, 1.06) | 0.060 | 0.89  (0.73, 1.09) | 0.150 | 1.00  (0.82, 1.23) | 0.926 | 0.98  (0.75, 1.28) | 0.914 |
| Canada (ref) vs. UK | **0.71**  **(0.58, 0.87)** | **<0.001** | 0.94  (0.75, 1.18) | 0.527 | **1.43**  **(1.17, 1.74)** | **<0.001** | 1.04  (0.86, 1.26) | 0.530 | **0.74**  **(0.59, 0.94)** | **0.001** |
| Mexico (ref) vs. US | 1.23  (0.99, 1.54) | 0.013 | 1.13  (0.90, 1.42) | 0.156 | 1.00  (0.82, 1.22) | 0.954 | **2.05**  **(1.70, 2.47)** | **<0.001** | 0.89  (0.67, 1.18) | 0.318 |
| Mexico (ref) vs. UK | 0.88  (0.72, 1.07) | 0.112 | **1.27**  **(1.01, 1.58)** | **0.005** | **1.61**  **(1.32, 1.96)** | **<0.001** | **2.13**  **(1.78, 2.54)** | **<0.001** | **0.67**  **(0.52, 0.87)** | **<0.001** |
| US (ref) vs. UK | **0.71**  **(0.58, 0.87)** | **<0.001** | 1.12  (0.90, 1.39) | 0.173 | **1.60**  **(1.32, 1.94)** | **<0.001** | 1.03  (0.85, 1.26) | 0.604 | **0.75**  **(0.59, 0.95)** | **0.002** |

**^1^RRR and 99% CI derived from multinomial logistic regression models adjusted for age, sex", race or ethnicity, education level, income adequacy and nutritional knowledge and weighted for post-stratification sampling weights. “Not available” was the reference category in all models.**
